# Chest Discomfort, Left Ventricular Hypertrophy, Global T‐Wave Inversion, and Short PR Interval Points to a Particular Cardiac Condition. What Could Be the Diagnosis?

**DOI:** 10.1111/anec.70048

**Published:** 2025-02-07

**Authors:** Jing‐Xiu Li, Xin‐Xin Di, Min Gao, Xue‐Qi Li, Yan‐Lin Wang, Jie Zheng

**Affiliations:** ^1^ Department of Electrocardiogram, The First Affiliated Hospital of USTC, Division of Life Sciences and Medicine, University of Science and Technology of China Hefei City Anhui China; ^2^ Department of Cardiology The Fourth Affiliated Hospital of Harbin Medical University Harbin China

**Keywords:** Fabry disease, left ventricular hypertrophy, Sokolow–Lyon index

## Abstract

This article describes a 44‐year‐old female with Fabry disease presenting with a 7‐year history of chest discomfort, extremity pain, and hypohidrosis. ECG revealed sinus bradycardia (52 bpm), a short PR interval (100 ms) with a delta wave, and a QRS complex (126 ms) showing a complete right bundle branch block. T‐wave inversion and ST‐segment depression were observed in leads I, AVL, II, aVF, and V2–V6. Genetic testing confirmed Fabry disease (GLA: c.700_702del). Short PR interval with left ventricular hypertrophy (LVH) poses a diagnostic challenge, requiring advanced imaging and genetic testing to differentiate Fabry disease from other cardiomyopathies.

## Graphical abstract

The co‐occurrence of short PR interval and left ventricular hypertrophy may indicate conditions such as Fabry disease, Danon disease, PRKAG2 mutation cardiomyopathy, Pompe disease, or mitochondrial disorders. This patient presents with symptoms of anhidrosis and chest discomfort. We identified one heterozygous mutation in exon 5 of GLA. Consequently, the diagnosis of FD was confirmed

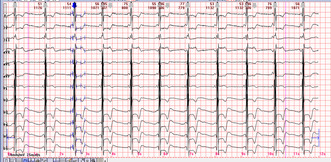

## 1 Case

**FIGURE 1:**
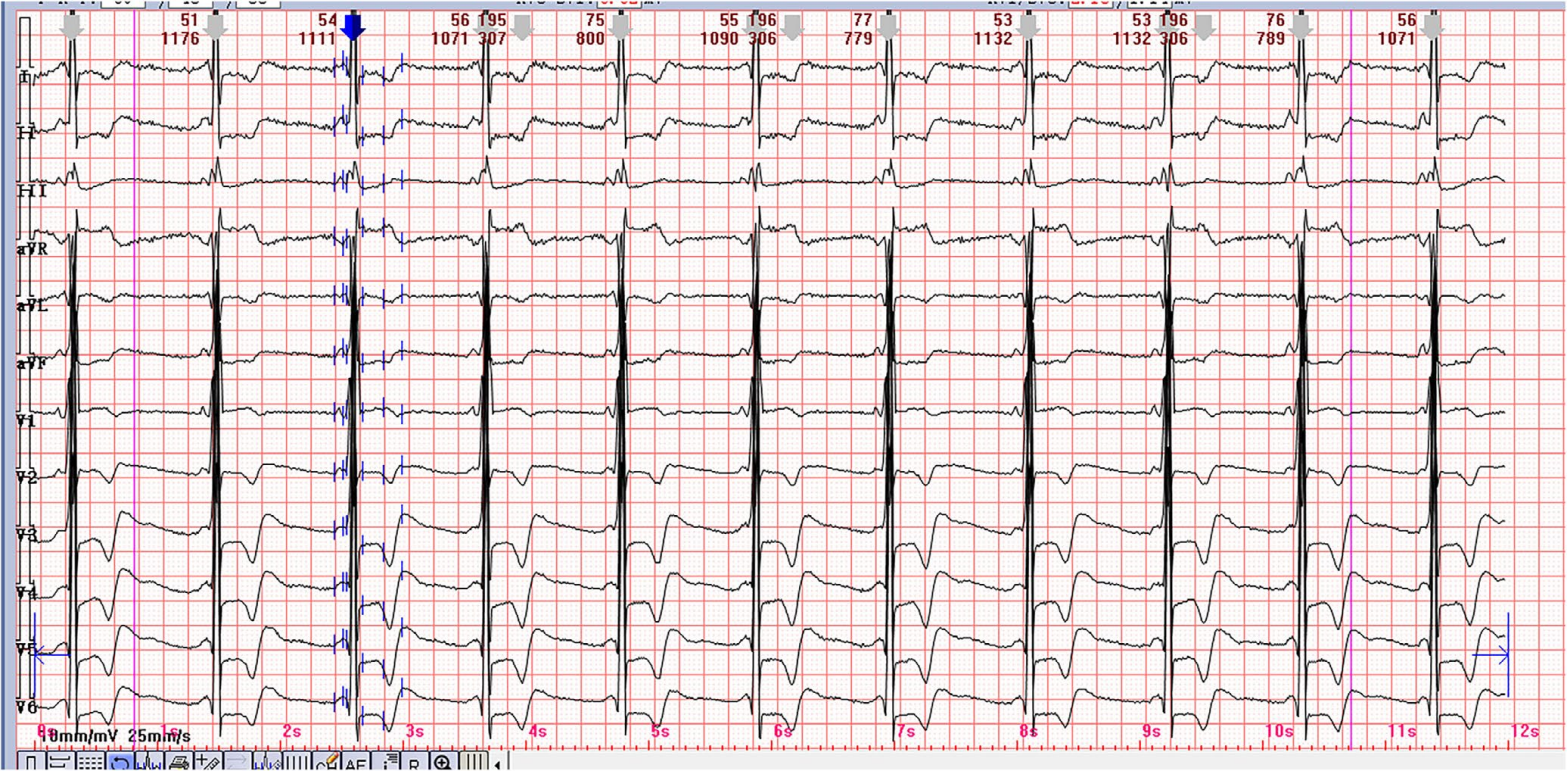
Twelve lead ECG on admission. The ECG displayed a sinus bradycardia with a heart rate of 52 beats per minute. The PR interval was short, measured at 100 milliseconds, accompanied by a delta wave. The QRS complex, with a duration of 126 milliseconds, exhibited a complete right bundle branch block pattern. The R‐wave amplitude recorded in lead V5 was 6.62 mV, the Sokolow–Lyon index (S in V1 + R in V5) was also 6.62 mV. T‐wave inversion and ST segment depression were noted in leads I, AVL, II, aVF, and V2–V6.

A 40‐year‐old patient presented with concerns about chest discomfort, episodic pain in the hands and feet, and hypohidrosis for 7 years. The patient had no history of hypertension or diabetes, and her vital signs were normal. Examination revealed a heart rate of 52 beats/min, and blood pressure was 122/81 mmHg. The electrocardiogram from the patient's admission is shown in Figure 1. The echocardiogram revealed an interventricular septum diameter (IVSD) of 11 mm and a left ventricular posterior wall diameter (LVPWD) of 12 mm. The left ventricular ejection fraction was recorded at 65%. Laboratory values were as follows: potassium, 4.09 mmol/L, creatine kinase‐MB 15 (0–25 IU/L), NT‐proBNP, 1160 pg/mL (0–100 pg/mL), D‐dimer 0.13 mg/L (0.01–0.50 μg/mL), urine protein +.

The ECG displayed a sinus bradycardia with a heart rate of 52 beats per minute. The PR interval was short, measured at 100 milliseconds, accompanied by a delta wave. The QRS complex, with a duration of 126 milliseconds, exhibited a complete right bundle branch block pattern. The R‐wave amplitude recorded in lead V5 was 6.62 mV, the Sokolow–Lyon index (S in V1 + R in V5) was also 6.62 mV. T‐wave inversion and ST‐segment depression were noted in leads I, AVL, II, aVF, and V2–V6. The provisional diagnosis was acute myocardial infarction. However, the patient's creatine kinase‐MB levels remained within normal limits. Therefore, the diagnosis of acute myocardial infarction was excluded. The contrast‐enhanced cardiac magnetic resonance imaging (CMR) demonstrated delayed gadolinium enhancement in the lateral region of the left ventricle. The Lyso‐GL‐3 level was 8.74 ng/mL (< 1.11 ng/mL). We identified one heterozygous mutation in exon 5 of GLA, GLA: ChrX: 100653872–100653874 NM_000169.3: c. 700_702 del (p.Asp 234 del). Therefore, it was confirmed that the condition was Fabry disease (FD). On the fifth day of hospitalization, the patient received enzyme replacement therapy. This treatment was well tolerated, with no reported adverse effects. The patient was subsequently discharged on the sixth hospital day.

## 2 Discussion

The ECG pattern is consistent with the Sokolow–Lyon criteria for diagnosing left ventricular hypertrophy (LVH) (Sokolow and Lyon 1949). Potential differential diagnoses include hypertensive heart disease, hypertrophic cardiomyopathy, physiological remodeling associated with an athletic heart and conditions presenting with HCM‐like manifestations (Gersh et al. 2011). The patient had no history of hypertension and was not involved in competitive athletics. The co‐occurrence of short PR interval and LVH may indicate conditions such as Fabry disease (FD), Danon disease, PRKAG2 mutation cardiomyopathy, Pompe disease, or mitochondrial disorders. This patient presents with symptoms of anhidrosis, proteinuria, chest discomfort, and episodic pain in the hands and feet. These findings should prompt consideration of FD as a possible diagnosis. We identified one heterozygous mutation in exon 5 of GLA. Consequently, the diagnosis of FD was confirmed.

FD is an X‐linked genetic disorder caused by mutations in the GLA gene, leading to a deficiency of the enzyme α‐galactosidase (Mehta et al. 2010). After individuals accumulate globotriaosylceramide and glycosphingolipids in the lysosomes and cytoplasm of cells, leading to multiorgan dysfunction of the cardiac, renal, skin, and cerebrovascular system. Electrocardiographic findings can provide crucial indicators of FD, encompassing a short PR interval, fulfillment of Sokolow voltage criteria for LVH, repolarization abnormalities, bundle branch block, atrioventricular conduction delay, progressive sinus node dysfunction, and arrhythmic manifestations including premature ventricular contractions. This patient exhibits high voltage in the left ventricle, alterations in repolarization as evidenced by changes in the ST segment and T wave, a short PR interval, a prominent delta wave, bundle branch block, and episodes of premature ventricular contractions. We pointed out that the delayed lateral endocardial gadolinium enhancement identified on the patient's MRI likely contributes to the extensive ST‐T segment alterations observed on the patient's ECG. Cardiac disease is linked to the buildup of globotriaosylceramide within all cellular elements of the heart, such as cardiomyocytes, cells of the conduction system, vascular fibroblasts, endothelial cells, and vascular smooth muscle cells (Mehta et al. 2010). In this Fabry disease patient, the pathological accumulation of glycosphingolipids within the cardiovascular system, particularly in small vessels and myocardial cells, underlies the occurrence of chest pain. This glycosphingolipid deposition leads to microvascular dysfunction, diminished coronary blood flow, and subsequent myocardial ischemia. Furthermore, associated cardiac remodeling, including left ventricular hypertrophy and fibrosis, contributes to impaired oxygen delivery and increased myocardial strain, thereby intensifying the likelihood of chest pain in these patients. Notably, the short PR interval is due to the accumulation of globotriaosylceramide in the atrioventricular node.

Medical professionals should be increasingly vigilant about differentiating hypertrophic cardiomyopathy from Fabry disease, as both conditions induce cardiac hypertrophy that appears similar to HCM but require distinct management approaches. Therefore, clinicians should recognize this ECG finding immediately and initiate appropriate treatment promptly as these measures may be vital in saving the patient's life.

## Author Contributions

Jing‐Xiu Li and Xue‐Qi Li contributed significantly to data collection and manuscript preparation. Xin‐Xin Di, Yan‐Lin Wang, Jie Zheng, and Min Gao performed the analysis with discussion. All authors agree on the order in which their names will be listed in the manuscript.

## Ethics Statement

We identify that the ethics committee of The First Affiliated Hospital of USTC has approved the case and that this case conforms to recognized standards, the Declaration of Helsinki.

## Conflicts of Interest

The authors declare no conflicts of interest.

## Data Availability

The authors have nothing to report.

## References

[anec70048-bib-0001] Gersh, B. J. , B. J. Maron , R. O. Bonow , et al. 2011. “2011 ACCF/AHA Guideline for the Diagnosis and Treatment of Hypertrophic Cardiomyopathy: A Report of the American College of Cardiology Foundation/American Heart Association Task Force on Practice Guidelines.” Circulation 124, no. 24: e783–e831. 10.1161/CIR.0b013e318223e2bd. 22068434

[anec70048-bib-0002] Mehta, A. , M. Beck , F. Eyskens , et al. 2010. “Fabry Disease: A Review of Current Management Strategies.” QJM 103, no. 9: 641–659. 10.1093/qjmed/hcq117. 20660166

[anec70048-bib-0003] Sokolow, M. , and T. P. Lyon . 1949. “The Ventricular Complex in Left Ventricular Hypertrophy as Obtained by Unipolar Precordial and Limb Leads.” American Heart Journal 37, no. 2: 161–186. 10.1016/0002-8703(49)90562-1. 18107386

